# Effect of Humidity-Controlled Dehydration on Microbial Growth and Quality Characteristics of Fresh Wet Noodles

**DOI:** 10.3390/foods10040844

**Published:** 2021-04-13

**Authors:** Jun-Jie Xing, Dong-Hui Jiang, Zhen Yang, Xiao-Na Guo, Ke-Xue Zhu

**Affiliations:** State Key Laboratory of Food Science and Technology, School of Food Science and Technology, Jiangnan University, Wuxi 214122, China; jjxing@jiangnan.edu.cn (J.-J.X.); jiangdonghui@163.com (D.-H.J.); zhen.yang@jiangnan.edu.cn (Z.Y.); xiaonaguo@jiangnan.edu.cn (X.-N.G.)

**Keywords:** fresh wet noodles, humidity-controlled dehydration, microorganisms, shelf-life, noodle quality

## Abstract

Humidity-controlled dehydration (HCD) was innovatively applied in this paper to control the growth of microorganisms in fresh wet noodles (FWN). Effects of HCD treatment with different temperatures (40, 60 or 80 °C), relative humidity (RH, 50%, 70% or 90%) and treatment time (5–32 min) on the total plate count (TPC), the shelf-life, and qualities of FWN were investigated. The results showed that HCD reduced the initial microbial load on the fresh noodles and extended the shelf-life up to 14–15 days under refrigeration temperature (10 °C). A 1.39 log_10_ CFU/g reduction for the initial TPC was achieved after HCD treatment at the temperature of 60 °C and RH of 90%. HCD with higher RH had a more positive influence on quality improvement. The L* values, the apparent stickiness, and the cooking properties of the noodle body were improved by HCD while good sensory and texture quality of noodles were still maintained after the dehydration process.

## 1. Introduction

Fresh wet noodles (FWN) are prone to be contaminated by microorganisms in production and distribution due to the high-water activity and rich nutrient content [1,2]. Therefore, the pasteurization of fresh noodles is always a key step to be assured in the industrial production of this traditional staple food, and it is also of great importance to effectively extend the shelf-life of noodle products without decreasing their edible quality [3]. Many studies have been done to adopt comprehensive measures to reduce microbial contamination by using raw materials with low bacteria load, controlling and improving sanitation during processing, packaging, and distribution of fresh noodle products [4,5,6].

A lot of research and attempt has been done for validation and improvement of the fresh-keeping techniques of FWN under room temperature. Decreasing pH by the addition of organic acid into fresh noodles had been proven to be an effective preservation method [7], while directly adding into or spraying edible alcohol on the surface of the noodles is currently the most widely used method for pasteurization during the fresh noodle production [8]. By adding a variety of natural and/or chemical preservatives, like sodium/calcium propionate, capryl monoglyceride, tea polyphenols, or chitosan into the noodle bodies, better antimicrobial and fresh-keeping results have been achieved in practice [5,9].

In addition to the above chemical methods, physical treatments, like heat and radiation could also kill microorganisms [6]. Most previous studies have been focused on non-thermal inactivation technologies including high hydrostatic pressure (HHP), pulsed electric field (PEF), intense pulsed light (IPL), low-temperature plasmas (LTP), ultrasound or ultraviolet light, etc. [1]. Other technologies like ozone water treatment, modified atmosphere packaging, control of water activity (a_w_), and hurdle technology had been tried for preservation purposes in terms of process improvement in the FWN field [10,11]. Moreover, heat treatment can destroy microorganisms by breaking the internal bonds of thermally activated molecules inside the organisms [12]. Although pasteurization by high-temperature heat treatment was reported not feasible for heat-sensitive food [1], moderate dehydration technology with high-temperature at 105–135 °C has been validated and applied in the production of Chinese semi-dried noodle to reduce the microbial load and the activity of the oxidase [2].

In general, the industrial drying process of noodle products was often at low temperature with divided steps, and the humidity of the hot air was usually taken into consideration for fine dried noodles, Udon noodles, and pasta products [13,14]. Empirically then, the relative humidity, as well as the temperature of the hot air, should be controlled during the drying process for both semi-dried noodles and fresh wet noodles. Nevertheless, heat treatment with excessive temperature and humidity may result in the over gelatinization or melting of starch, the generation of cracks or chaps in fresh noodles, thus deteriorating the noodle quality [15]. In terms of microbial control and quality improvements, it is hypothesized that the fresh noodles would benefit a lot from heat treatment with moderate or mild conditions. We, therefore, attempted a trial of thermal treatments for fresh noodles with a gentler method by employing humidity-controlled hot air to test that hypothesis [16]. And to the best of our knowledge, the method has not been done yet. Therefore, this study aims to investigate the effects of thermal dehydration treatment with medium temperature and controlled RH on the shelf-life and qualities of the fresh wet noodles.

## 2. Materials and Methods

### 2.1. Materials

Wheat flour was obtained from Yihai Kerry Grain and Oil Industry, and the protein, ash, and moisture contents were 11.28 ± 0.15%, 0.44 ± 0.09%, and 13.18 ± 0.07%, respectively. The table salt used in the noodles was from the local supermarket. Other reagents used were of analytical grade.

### 2.2. Preparation of the Fresh Wet Noodles (FWN)

The formula of the fresh wet noodle in this study consisted of 1000 g of flour, 340 mL distilled water, and 10 g NaCl. For noodle processing, the flour, distilled water, and NaCl were put into a vacuum mixer (Model HWJZ-5, Nanjing, China) and mixed for 7 min under a vacuum degree of −0.08 MPa to form the noodle dough. The dough was put into a plastic bag that was sterilized by ultraviolet radiation beforehand and then rested for 25 min at 25 °C. Then, the dough was passed through a small roller noodle machine (Model JMTD-168/140, Beijing, China) gradually. The thickness was reduced from 2 mm to 1.5 mm with the roller gap and finally reduced to 1 mm to obtain a dough sheet. The dough sheet was passed through the noodle machine to get the resultant noodle strands with the dimensions of 1.0 mm in both width and thickness. The water content of the noodle body was about 34% after noodle preparation.

### 2.3. Humidity-Controlled Dehydration (HCD)

The above fresh noodles were thermally treated by using automatic noodle drying equipment (SYT-030, Beijing, China). The dehydration temperature and relative humidity were adjusted to 40, 60, 80 °C, and 50%, 70%, 90% with different combinations before operations. In this paper, the noodle sample treated under the temperature of 40 °C and RH of 50% was abbreviated as sample “40 °C-50%” and so on. Since the dehydration speed varied according to the thermal treatments, the dehydration time was different depending on the conditions when the water content of all the noodle samples was reduced from 34% to the same level (about 30%). The moisture content variations and the corresponding dehydration time of the HCD treated noodles were listed in Table 1 and the water content of the control sample was 34.53%. The obtained HCD noodles were cooled at room temperature (about 25 °C) for 1 h in a sterilized plastic bag and then the sealed noodles were stored at 10 °C before the microbial analysis.

### 2.4. Determination of Bacterial Content

The HCD treated noodles (25 g) in different storage stages were sampled, pulverized, and mixed with 225 mL of 0.85% aseptic physiological saline. The mixture was homogenized by a stomacher machine (Lab-blender 400; Seward Laboratory) for 60 s before transferred to make different series of dilutions using 0.85% aseptic physiological saline. 1 mL of appropriate dilutions was pipetted onto sterile plate count agar plates and incubated at 36 °C for 48 ± 2 h before the examination of the total plate counts (TPC) according to GB/T 4789.2-2016 [17]. The yeasts and molds count (YMC) were also calculated using Bengal red medium after incubation for 5 days at 28 °C according to GB/T 4789.15-2016 [18].

### 2.5. Color Measurement of the HCD Noodles

The change of L* value of the HCD treated noodles in different storage days were measured by a Chroma Meter (Konica Minolta CR-400, Osaka, Japan) equipped with a D65 illuminant. Ten thin strips of the noodles were closely arrayed as a line, and measurement was carried out at 4 testing points on the surface of each noodle sample. L* is a measurement of noodle brightness (100 = white, 0 = black) according to CIE L*, a*, and b* system, and each of the sample points was tested three times.

### 2.6. Determination of the Degree of Gelatinization

The degree of gelatinization in the noodles was measured based on the starch-iodine complexes reaction, according to the method reported by Wootton et al. [19]. The HCD noodles were lyophilized, ground, and passed through a 100 mesh sieve. Two treated samples (1 g) were placed in tube 1 and tube 2, and the negative control in tube 1, respectively. Distilled water (50 mL) was then added to each tube, followed by shaking. The sample in tube 1 was heated in a boiling water bath for 20 min and then cooled to room temperature. Glucoamylase (2 mL) was added to each tube and incubated in a water bath at 50 °C for 1 h with shaking every 15 min. After 1 h of incubation, 2 mL of HCl (1 M) was added into each tube to terminate the reaction. Each sample was placed in a 100 mL volumetric flask filled with distilled water and filtered. The filtrate (10 mL) of each diluted sample was placed in a 250 mL volumetric flask, and 10 mL of iodine solution (0.1 M) and 18 mL of NaOH were added. The flask was then sealed for 15 min before the addition of 2 mL of sulfuric acid (10%, w/w). Sodium thiosulfate (0.1 M) was used as an indicator to determine the endpoint of titration and the volume consumption was recorded. The degree of gelatinization was calculated by the equation:

Degree of gelatinization (%) = (*V*_0_ − *V*_2_)/(*V*_0_ − *V*_1_) × 100

where *V*_0_ is the volume (mL) of sodium thiosulfate consumed by a blank sample, *V*_1_ is the volume (mL) of sodium thiosulfate consumed by a fully gelatinized noodle sample, *V*_2_ is the volume (mL) of sodium thiosulfate consumed by the ungelatinized sample.

### 2.7. Cooking and Textural Properties of the Fresh Wet Noodles

#### 2.7.1. Cooking Loss and Water Absorption Index

The cooking loss of the HCD noodles was determined as described by AACC Method 66–50 [20]. The raw noodle sample (Ws, about 25 g) was placed into 400 mL of boiling distilled water until the optimal cooking time (OCT) was reached. The boiled noodle was removed from the cooking water and drained for 3 min and weighed. The cooking water was cooled to room temperature and transferred in a 500-mL volumetric flask filled with distilled water, and then 100 mL of it was transferred to a 250-mL beaker. After that, the beaker was placed into an air oven to dry at 105 °C until a constant weight was reached. The residue was weighed and calculated as a percentage of the starting material (dry weight basis). In a separate test, the same amount of fresh noodles was weighted, cooked to OCT, and removed from the cooking water. The boiled noodle was then reweighted after absorbing the water on the surface with 5 layers of filter paper. The water absorption index was calculated as the ratio of cooked noodle weight over the dry sample weight.

#### 2.7.2. Textural Properties

The texture properties of the uncooked and cooked HCD noodles were determined using a TA-XT2i texture analyzer (Stable Micro Systems, London, UK). The P/35 probe was used to measure the apparent stickiness of uncooked HCD noodles. The hardness of cooked HCD noodles was measured according to the description of Wu et al. with some modifications [21]. Fresh wet noodles were cooked to the OCT before removed from the cooking water and the remaining moisture on the surface of the boiled noodles was quickly dried by using five layers of filter paper after draining with cold water. Testing was completed within 15 min. The tensile testing was performed with an A/SPR probe and one strand of noodle was located through slots and was wound round parallel friction roller two or three times. The distance between the two rollers was set at 50 mm and the maximum operation distance was 100 mm. The pretest and test speed were 2.0 mm/s, and the post-test speed was 10.0 mm/s. For the hardness test, three strands of noodles were arrayed on the platform with HDP/FPS probe, and the pretest, test, post-test speed were 2, 0.8, 0.8 mm/s, respectively. The trigger force was 5 g and the compressive strain was 75%. The pause between the first and second compression was 2 s. Each of the noodle samples was tested at least 7 times and an average was applied.

### 2.8. Sensory Evaluation

The uncooked and cooked HCD fresh noodles were prepared for sensory evaluation using quantitative descriptive analysis (QDA) according to the method of Costa et al. [22]. The sensory evaluation panel consisted of 12 trained tasters (8 females and 4 males). The uncooked noodle samples were submitted to the panelists for estimation of color and appearance. A ten-point hedonic rating scale with “10” indicating “like extremely” and “1” indicating “dislike extremely”. The chewiness (“20”), elasticity (“25”), adhesiveness (“15”), smoothness (“5”), flavor (“5”), and overall acceptability (“10”) were assigned different weights and evaluated for the cooked noodles. The sensory tests were conducted in triplicate.

### 2.9. Scanning Electron Microscopy

The morphology of fresh wet noodles after the HCD treatment under different conditions was investigated using a scanning electron microscope (Quanta-200; FEI Ltd., Eindhoven, The Netherlands). Noodle samples were rinsed, eluted, and freeze-dried. The samples were then mounted with double-sided carbon adhesive tabs on aluminum stubs, and coated with gold-palladium. The surface and cross-section of the noodles were observed and photographed at an accelerating voltage of 1.0 kV.

### 2.10. Statistical Analysis

All of the above-mentioned tests were carried out in triplicate and the results are expressed as the mean ± standard deviation. SPSS 17.0 software (SPSS Inc., Chicago, CA, USA) was used for the analysis of variance (ANOVA), and a significant difference between any two means was calculated by using Duncan’s multiple range tests. A 0.95 confidence level (*p* < 0.05) was applied to determine if a significant difference existed between the two mean values.

## 3. Results and Discussion

### 3.1. Microbial Analysis of HCD Fresh Wet Noodles

#### 3.1.1. Initial Bacterial Content

**Figure 1:**
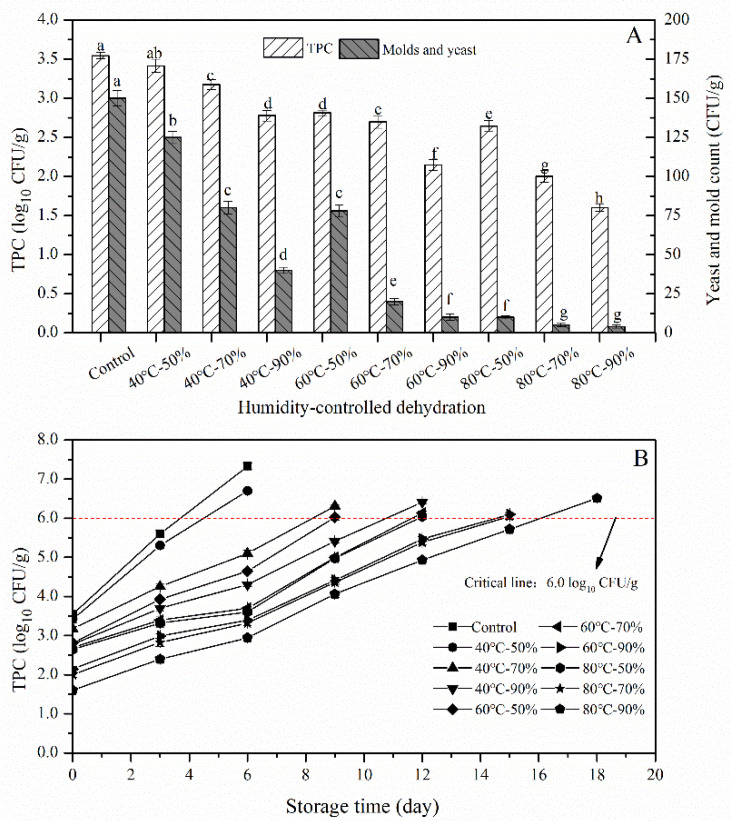
Application of humidity-controlled dehydration (HCD) in microbial control of fresh noodles. (**A**), effect of HCD on the initial total plate count (TPC) and yeast and mold count (YMC). (**B**), effect of HCD on the microorganism growth of fresh noodles during storage. Control, fresh wet noodles; 40 °C-50%, fresh wet noodle samples treated by humidity-controlled dehydration under the condition of 40 °C and relative humidity (RH) of 50%, etc. The different lowercase letter means there was a significant difference in TPC/YMC (*p* < 0.05).

The effect of HCD on the initial bacterial content including TPC and YMC was shown in Figure 1A. Under the same condition of temperature or relative humidity, the initial TPC of HCD noodle samples was significantly decreased (*p* < 0.05) with the increase of relative humidity or temperature, respectively. In this study, the moisture content of fresh wet noodles was settled to the same level of 30% to evaluate the effect of HCD on storage quality of fresh wet noodles. Therefore, the RH and HCD time would depend on each other, since, at higher RH, the time needed to dry the product is longer due to the lower moisture gradient between product and air. This result indicated that HCD could inhibit the growth of microbes to a certain degree, and it had a more positive effect under the condition of either higher RH and temperatures, or longer HCD time. By comparison, the initial TPC of 40 °C-70%, 60 °C-70% and 80 °C-70% samples were lower than that of the control by 0.37, 0.85, 1.54 log_10_ CFU/g, respectively, and a maximum inactivation level of 1.94 log_10_ CFU/g for the initial TPC of the HCD noodles was achieved in this study under the conditions of 80 °C-90%. As mentioned by Chau et al. [12], heat treatment in a high-temperature gas or environment could destroy the microorganism by activating the molecules of the organism and breaking down their internal bonds. In our previous study, the shelf-life of semi-dried noodles was extended 5 days after high-temperature-short-time (HTST, 120 °C) dehydration treatment, and the TPC was decreased significantly [2]. Inspired by this work, we tried to explore the applicability of thermal dehydration technology in the fresh wet noodles field, with some modifications. The results agreed with the previous study and suggested some thermolabile bacteria were inactivated during the HCD process and the relative humidity could affect the efficiency of the inactivation by heat. For instance, 60 °C of HCD significantly decreased the initial TPC of fresh wet noodles by 20.7, 23.9, and 39.5 percent as RH increased from 50% to 70% and 90%. It should be noted that the HCD time was also increased accordingly from 5 to 23 min, therefore, the effect of RH should be discussed in conjunction with the effects of HCD time. The YMC of the fresh noodles was reduced from 150 CFU/g to 125, 78, and drastically to 10 CFU/g as the temperature increased from 40 to 60 and 80 °C under the same RH of 50% (Table 1). All yeasts and molds have their optimal growth temperature: that is, if the temperature was raised above a certain point or critical point, most of the yeasts and molds would be killed by thermal treatment. The prime example was the progress of high-temperature short-time (HTST) pasteurization technology in the milk field, and most of the potentially harmful bacteria could be killed by increasing the temperature to about 60–80 °C [23,24]. In this study, the yeasts and molds in fresh noodles were effectively inactivated at 80 °C and the removal efficiency was above 90% (which was 1-log_10_ reduction) after 3 min of thermal dehydration. All the microorganisms, no matter it is spoilage or pathogenic bacteria, prefer to grow in high-a_w_ conditions and some foodborne pathogens were able to survive in foods with low a_w_ as well as in dry environments [25,26]. Except for the temperature, the relative humidity also had a great effect on the microbial thermo-tolerance in thermal treatment [26]. The results showed almost 50% of yeasts and molds were induced to death by thermal treatment at 60 °C and 50% of RH, while a higher inactivation rate of 90% (1-log_10_ reduction) was achieved for yeasts and molds in FWN when the RH was increased to 90%. A high level of relative humidity in environments should have led to a large number of microbes growing, but the high-moisture hot air during HCD induced microbial cell killing instead. Therefore, HCD with higher RH (longer HCD time) had a more positive influence on reducing both TPC and YMC, and we speculated that some heat-resistant bacteria could be inactivated under high temperatures with higher humidity.

#### 3.1.2. Microbial Growth during Storage

According to the report of Li et al. [2], the fresh wet noodles would deteriorate and their quality lowered once the TPC was over 10^6^ CFU/g and hence the quality analysis of noodle samples would be terminated in this study [11]. As shown in Figure 1B, the microbial load of the control sample with an initial TPC of 3.54 log_10_ CFU/g would quickly exceed the threshold level in 3–4 days during storage at 10 °C, and soon the fresh wet noodles would deteriorate. After HCD treatment, the shelf-life of FWN was extended up to 9–15 days with the increase of treating temperature and RH. The initial TPC is one of the most important factors that affect the shelf-life of fresh wet noodles, and in general, the shelf-life of FWN with a lower initial bacterial load could be prolonged. After HCD treatment, the initial TPC of sample 60 °C-90% was reduced by 1.39 log_10_ CFU/g compared with the control, and the shelf-life of which was extended up to more than 12 days. Since there was no significant difference (*p* > 0.05) in initial TPC between the sample 40 °C-90% and 60 °C-50%, and between the sample 60 °C-70% and 80 °C-50%, we could not distinguish the corresponding samples (with same water content of 30%) in terms of TPC variations and shelf-life.

The change of YMC of FWN during storage was similar to that of TPC and was listed in Table 1. HCD also had a great influence on the growth of yeasts and molds. Based on our experience, we assumed that the fresh noodles would deteriorate quickly after 3 days of storage if the YMC exceeded 2000 CFU/g, which was in step with the evolution of TPC. Therefore, it is important to take measures to reduce the initial microbial load to extend the shelf-life of FWN.

### 3.2. Quality Analysis of HCD Fresh Wet Noodles

#### 3.2.1. Color Changes

**Figure 2:**
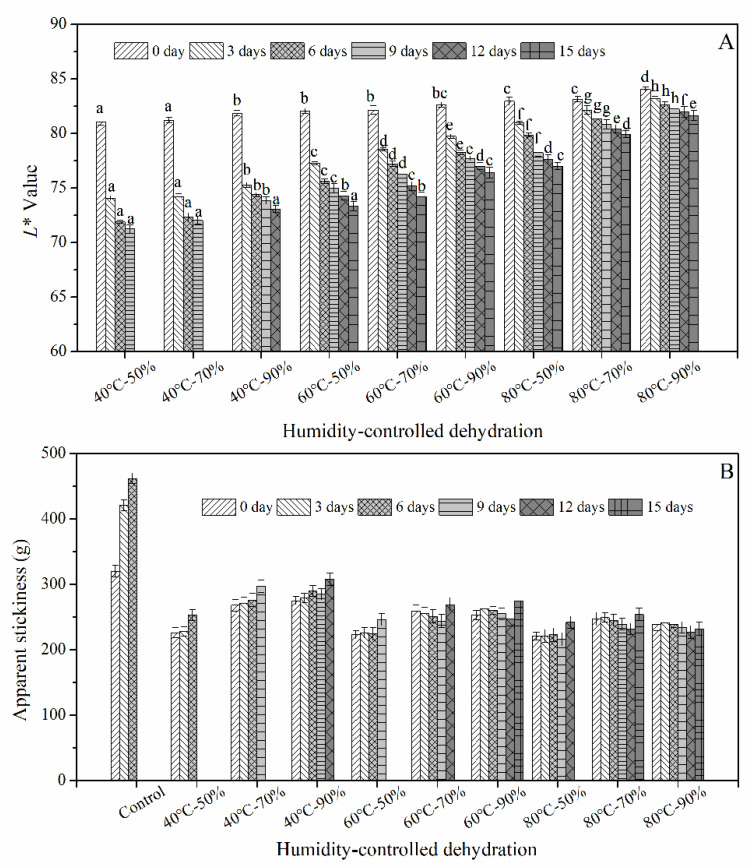
Effect of humidity-controlled dehydration on the L* value (**A**) and apparent stickiness (**B**) of fresh wet noodles during storage. Control, fresh wet noodles; 40 °C-50%, fresh wet noodle samples treated by humidity-controlled dehydration under the condition of 40 °C and RH of 50%, etc. The different lowercase letter means there was a significant difference in the same storage time (*p* < 0.05).

Fresh wet noodles are prone to enzymatic and non-enzymatic browning during storage [2]; this would result in the noodle darkening and could be measured by the change of L* value during storage (Figure 2A). Although excessive heat treatment has been reported to deteriorate the noodle quality including color degradation [26], the L* value of HCD noodles in this study was much higher than the control (about 76, data not shown) and were increased to some extent with the increase of dehydration temperature which indicated a brighter color. This was mainly due to that moderate thermal treatment could inactivate enzymes especially the polyphenol oxidase [24]. The decrease in L* value (Δ*L*) was 6.95 after 3 d of storage for sample 40 °C-50%, which was only 0.83 for sample 80 °C-90%. For HCD samples treated at 60 and 80 °C, the change of L* value during storage was less with increasing relative humidity from 50% to 90% since the dehydration time was accordingly extended from 3–5 min to 18–23 min, respectively (Table 1). The experiment also showed that the color change was consistent with the results when applying HTST dehydration on semi-dried noodles [2]. The browning rate was slowed down by HCD treatment in this study, which was of great significance to reduce the color change during the storage of fresh noodles [27].

#### 3.2.2. Apparent Stickiness

As shown in Figure 2B, the apparent stickiness of fresh noodles was significantly decreased after the HCD treatment; this was mainly because the water evaporation on the surface of noodle bodies would result in the formation of a hard shell, thus the stickiness decreased. In general, the stickiness was decreased a little bit with the increase of dehydration temperature and increased with the increase of the relative humidity. Particularly, the apparent stickiness of the sample group (60 and 80 °C) was reduced and the increase of which was slowed down during storage. This may due to the aggregation of protein during the HCD treatment. The aggregated protein network would reduce the starch swelling, making the fresh wet noodles firmer and non-sticky [28]. This would be discussed in the next part based on the changes in the degree of gelatinization.

#### 3.2.3. Degree of Gelatinization

The changes in the degree of gelatinization of the HCD fresh wet noodles were listed in Table 1. During the dehydration process, the starch in fresh noodles gelatinized when the temperature increased up to 60–80 °C. Besides temperature, the degree of gelatinization of the HCD noodles was also influenced by the relative humidity, since it needs more time to dehydrate under higher humidity, during which gelatinization may happen. However, a relatively high degree of gelatinization would make the characteristics of fresh noodles deviate from the characteristic of the raw noodles. And through the analysis of sensory evaluation of fresh noodles with different gelatinization degrees in preliminary experiments, the level of 35% could be considered as the acceptable threshold of starch gelatinization degree for fresh noodles. As listed in Table 1, there was no significant difference in gelatinization degree between and among the 40 °C sample groups and the control fresh noodles. With the increasing temperature at 60 and 80 °C, the gelatinization degreed of HCD noodles was increased up to 33.83% for the sample 80 °C-90%. This is mainly because the final water amount remaining in the HCD fresh wet noodles was about 30% and might not be sufficient to cause complete swelling and starch gelatinization [27]. On the other hand, the gluten matrix in the noodles was capable of entrapping starch granules and limiting their swelling [27]. Therefore, the HCD noodles in this study still possessed the characteristic of raw fresh noodles.

#### 3.2.4. Microstructure

**Figure 3:**
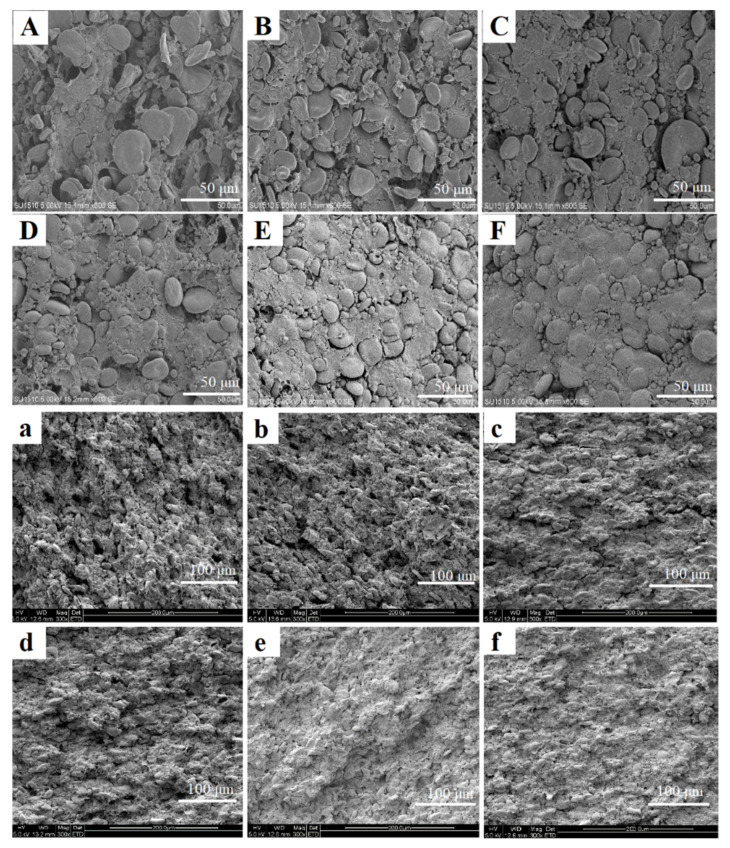
Microstructure changes of fresh noodles after humidity-controlled dehydration (HCD) treatment. (**A**–**F**), surface of HCD noodles with 40 °C-50%, 40 °C-90%, 60 °C-50%, 60 °C-90%, 80 °C-50%, 80 °C-90%; (**a**–**f**), cross sections of HCD noodles with 40 °C-50%, 40 °C-90%, 60 °C-50%, 60 °C-90%, 80 °C-50%, 80 °C-90%.

The microstructure of the fresh wet noodles treated with HCD under different conditions was investigated by scanning electron microscope (SEM). The micrographs of the surface (Figure 3A–F) and the cross-section (Figure 3a–f) part of the noodles in 600× and 300× magnification were shown in Figure 3. The obtained micrograph revealed that the 40 °C-50% HCD noodles presented a dispersed or incompact gluten network formation with sphere-shaped starch granules, which did not swell too much (similar to the control, figure not shown). The noodle surface gradually turned from initially rough into smooth as the temperature and RH rose to 60 °C and 90%, respectively, and the cross-sections turned from porous into compact. For the sample 80 °C-90%, the starch granules were observed to be encapsulated by protein and a dense and continuous network was formed, which is required for excellent cooking and edible quality.

### 3.3. Cooking and Texture Properties of the HCD Fresh Wet Noodles

**Figure 4:**
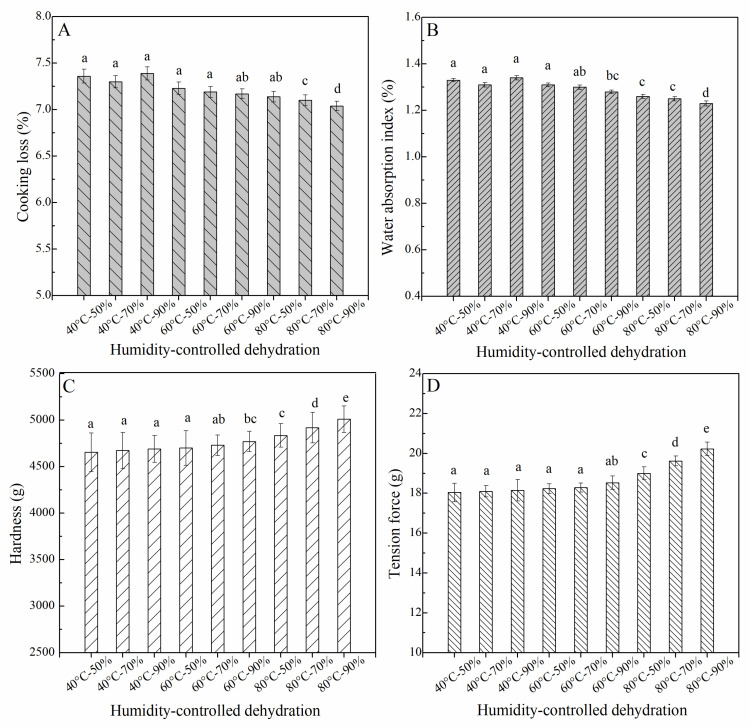
Effect of humidity-controlled dehydration on the textural and cooking quality of fresh wet noodles. (**A**), hardness; (**B**), tension force; (**C**), cooking loss; (**D**), water absorption index. 40 °-50%, fresh wet noodle samples treated by humidity-controlled dehydration under the condition of 40 °C and RH of 50%, etc.

Maintaining integrity during cooking is crucially important for improving noodle quality [21]. After the HCD treatment, both cooking loss and water absorption of the HCD fresh wet noodles were decreased with increasing temperature and RH (Figure 4A,B). This indicated that the leaching of amylose and the dissolution of the water-soluble protein during boiling was restrained to some extent by HCD [2]. And the cooking loss and water absorption index of the 40 °C group samples were almost the same, and there was no statistically significant difference between and among samples. Only when the dehydration temperature increased to 80 °C, the cooking parameters of corresponding noodle samples would show a decrease with increasing relative humidity. The cooking quality could be highly affected by starch gelatinization and protein aggregation [27]. Baiano et al. [29] has reported that high temperature during the dehydration process could induce the protein aggregation in fresh noodles, and the dense gluten network would entrap the starch granules, and led to the reduced swelling of the starch granules and leaching of the starch molecules during cooking [2,28].

The tensile force and hardness of the cooked HCD noodles were primarily affected by the dehydration temperature, and then the relative humidity. Wagner et al. [28] have reported that disulfide-sulfhydryl (SH) would exchange for glutenin and gliadins when the temperature was above 55 and 70 °C, respectively. At the same time, starch gelatinization may happen during the HCD process at 60 or 80 °C. Therefore, the cooking and textural properties of the fresh noodles after humidity-controlled dehydration treatment in this study were improved and superior to those of the control [27,28].

### 3.4. Sensory Evaluation of HCD Fresh Wet Noodles

Changes in sensory properties for uncooked and cooked HCD noodles were listed in Table 2. Each indicator was assigned a corresponding weight based on its importance in meeting the demands of consumers. Overall, the fresh wet noodles without dehydration process scored the highest of 93.48 in almost all indicators except that the color and the total score of the sensory assessment for HCD noodles fell between 88.36 and 89.48. However, there was no significant difference in adhesiveness and smoothness among and between the HCD noodle samples and the control; little difference in other indicators among the 40, 60, or 80 °C sample groups. On the other hand, although there was no statistical difference between the 40 °C sample group and the control, both the color and flavor of FWN were deteriorated as the dehydration temperature increased to 60 and 80 °C. There is a strong correlation between the appearance and the overall acceptability and between chewiness and toughness of the fresh noodles; meanwhile, dehydration at middle temperature (60–80 °C) is more likely to give good results than at low temperature (40 °C). Heat treatment could induce changes in protein network formation and starch gelatinization in fresh noodle bodies, thus affects the cooking, textural, and sensory properties of cooked noodles [27]. Although various aspects of the sensory properties of FWN were not affected too much by HCD treatment, to some extent, this is just what we need. In conclusion, the fresh noodles could still maintain the characteristics of raw noodles in terms of sensory evaluation after humidity-controlled dehydration treatment, which was developed mainly for the control of microbial safety purposes.

## 4. Conclusions

In this study, thermal dehydration with humidity-controlled hot air was innovatively applied as an effective way to control the initial microbial load in the fresh wet noodles before storage. HCD treatment could significantly reduce the microbial load in noodle bodies and prolong the shelf-life. The storage stability of color and apparent stickiness of the FWN was greatly improved after the humidity-controlled dehydration process, meanwhile, the textual and cooking properties also benefited a lot from the process. The humidity-controlled dehydration treatment seemed like a promising technique to pretreat the fresh wet noodles before packaging and distribution. However, further research is needed to optimize the conditions depending on the water content added in practical production when considering the economic benefits.

## Figures and Tables

**Table 1:** Humidity-controlled dehydration (HCD) and its effect on the final moisture content, gelatinization degree, and the changes of yeasts and molds count of fresh wet noodles *.

HCD			Final Moisture Content (%)	Gelatinization Degree (%)	Yeasts and Molds Count at Different Storage Time (CFU/g)						
Temperature (°C)	RH (%)	Time (min)			0 day	3 days	6 days	9 days	12 days	15 days	18 days
Control			34.00 ± 0.15 ^a^	23.14 ± 0.74 ^a^	150	2000	5000	-	-	-	-
40	50	6	30.18 ± 0.20 ^a^	23.46 ± 0.65 ^a^	125	1450	3800	-	-	-	-
	70	12	30.36 ± 0.19 ^a^	23.63 ± 0.71 ^a^	80	350	1750	3200	-	-	-
	90	32	30.27 ± 0.20 ^a^	23.81 ± 0.47 ^a^	42	200	1280	2200	-	-	-
60	50	5	30.21 ± 0.22 ^a^	24.66 ± 0.62 ^ab^	78	190	1120	2000	-	-	-
	70	10	30.33 ± 0.17 ^a^	25.71 ± 0.38 ^b^	20	25	65	310	1550	-	-
	90	23	30.25 ± 0.21 ^a^	27.33 ± 0.41 ^c^	10	10	10	20	20	400	-
80	50	3	30.38 ± 0.16 ^a^	31.39 ± 0.42 ^d^	10	10	10	15	350	1800	-
	70	7	30.29 ± 0.24 ^a^	32.04 ± 0.53 ^de^	<10	<10	<10	<10	<10	<10	20
	90	18	30.25 ± 0.23 ^a^	33.83 ± 0.55 ^e^	<10	<10	<10	<10	<10	<10	20

*, Values of moisture content represent mean ± S.D., *n* = 3. RH, relative humidity; The same lowercase letter means there was no significant difference within the same column; -, test was terminated by human intervention; <10, not detected or below the detectable limit.

**Table 2:** Effect of humidity-controlled dehydration (HCD) on the sensory evaluation of uncooked and cooked fresh wheat noodles *.

HCD	Value of Sensory Evaluation								
	Uncooked Noodles		Cooked Noodle Samples						
	Color	Appearance	Chewiness	Elasticity	Adhesiveness	Smoothness	Flavor	Overall Acceptability	Total Score
Control	9.70 ± 0.13 ^a^	9.69 ± 0.19 ^a^	18.79 ± 0.18 ^a^	23.42 ± 0.23 ^a^	13.72 ± 0.18 ^a^	4.27 ± 0.18 ^a^	4.71 ± 0.14 ^a^	9.18 ± 0.15 ^a^	93.48
40 °C-50%	9.83 ± 0.16 ^a^	9.06 ± 0.19 ^b^	17.20 ± 0.15 ^c^	21.28 ± 0.20 ^c^	13.70 ± 0.17 ^a^	4.24 ± 0.21 ^a^	4.46 ± 0.27 ^a^	8.59 ± 0.18 ^b^	88.36
40 °C-70%	9.79 ± 0.19 ^a^	9.04 ± 0.17 ^b^	17.18 ± 0.20 ^c^	21.55 ± 0.18 ^c^	13.71 ± 0.20 ^a^	4.21 ± 0.23 ^a^	4.44 ± 0.21 ^a^	8.63 ± 0.22 ^b^	88.55
40 °C-90%	9.78 ± 0.23 ^a^	9.11 ± 0.22 ^b^	17.22 ± 0.21 ^c^	21.49 ± 0.23 ^c^	13.71 ± 0.22 ^a^	4.26 ± 0.24 ^a^	4.17 ± 0.28 ^b^	8.67 ± 0.24 ^b^	88.41
60 °C-50%	9.49 ± 0.16 ^b^	9.24 ± 0.24 ^b^	17.35 ± 0.25 ^b^	22.27 ± 0.24 ^b^	13.71 ± 0.18 ^a^	4.23 ± 0.26 ^a^	4.05 ± 0.20 ^b^	8.78 ± 0.15 ^b^	89.12
60 °C-70%	9.42 ± 0.22 ^b^	9.30 ± 0.29 ^a^	17.47 ± 0.23 ^b^	22.11 ± 0.19 ^b^	13.69 ± 0.24 ^a^	4.22 ± 0.18 ^a^	3.94 ± 0.21 ^b^	8.84 ± 0.27 ^a^	88.99
60 °C-90%	9.43 ± 0.17 ^b^	9.37 ± 0.26 ^a^	17.45 ± 0.18 ^b^	22.30 ± 0.21 ^b^	13.70 ± 0.17 ^a^	4.29 ± 0.25 ^a^	3.90 ± 0.23 ^b^	8.88 ± 0.27 ^a^	89.32
80 °C-50%	9.48 ± 0.28 ^ab^	9.41 ± 0.20 ^a^	17.56 ± 0.17 ^b^	22.05 ± 0.24 ^b^	13.73 ± 0.16 ^a^	4.25 ± 0.26 ^a^	3.95 ± 0.17 ^b^	8.81 ± 0.21 ^a^	89.24
80 °C-70%	9.43 ± 0.17 ^b^	9.39 ± 0.30 ^a^	17.62 ± 0.19 ^b^	22.25 ± 0.26 ^b^	13.71 ± 0.25 ^a^	4.25 ± 0.18 ^a^	3.91 ± 0.18 ^b^	8.92 ± 0.19 ^a^	89.48
80 °C-90%	9.41 ± 0.16 ^b^	9.42 ± 0.18 ^a^	17.09 ± 0.20 ^c^	22.18 ± 0.28 ^b^	13.69 ± 0.15 ^a^	4.29 ± 0.26 ^a^	3.86 ± 0.16 ^b^	8.62 ± 0.23 ^b^	88.56

*, Control, fresh wheat noodles; 40 °C-50%, fresh wet noodles were treated by humidity-controlled dehydration under the conditions of 40 °C and RH of 50%, etc. Values represent mean ± S.D., *n* = 3. The same lowercase letter means there was no significant difference within the same column.

## Data Availability

The data that support the findings of this study are available on request from the corresponding author. The data are not publicly available due to privacy or ethical restrictions.
